# Chemical Compounds Identification and Antioxidant and Calcium Oxalate Anticrystallization Activities of *Punica granatum* L.

**DOI:** 10.1155/2020/9424510

**Published:** 2020-02-24

**Authors:** Rabie Kachkoul, Tarik Squalli Houssaini, Mohamed Mohim, Radouane El Habbani, Anissa Lahrichi

**Affiliations:** ^1^Laboratory of Biochemistry, Faculty of Medicine and Pharmacy, Sidi Mohammed Ben Abdellah University, BP 1893, Km 22, Road of Sidi Harazem, Fez, Morocco; ^2^Faculty of Science and Technology, Sidi Mohammed Ben Abdellah University, BP 2202, Road of Imouzzer, Fez, Morocco; ^3^Laboratory of Molecular Bases in Human Pathology and Therapeutic Tools, Faculty of Medicine and Pharmacy, Sidi Mohammed Ben Abdellah University, BP 1893, Km 22, Road of Sidi Harazem, Fez, Morocco; ^4^Department of Nephrology, University of Hospital Hassan II, BP 1835, Atlas, Road of Sidi Harazem, Fez, Morocco

## Abstract

The plant *Punica granatum* L. has several biological activities and a great curative and preventive power against chronic diseases. For this purpose, the objective of this work is to valorize the fruit peel of this plant in the field of phytomedicine, by quantifying and identifying its bioactive compounds and by evaluating their antioxidant and anticrystallization activities against calcium oxalate. This comparative study has been carried out by hydroalcoholic extract (E.PG) and infusion (I.PG) of the plant. The quantification of the phenolic compounds has been performed by spectrophotometric methods, and the chemical species identification has been performed by UPLC-PDA-ESI-MS. Moreover, the examination of the antioxidant activity has been executed by both methods of DPPH and FRAP. The crystallization inhibition has been studied in vitro by the turbidimetric model. The characterization of the synthesized crystals has been accomplished by microscopic observation and by Fourier Transform Infrared Spectroscopy. The results found show the comparable importance of the two plant extracts in the elimination of free radicals; the values of the half maximal inhibitory concentration “IC_50_” obtained are in the order of 60.87 ± 0.27 and 59.91 ± 0.83 *μ*g/mL by the DPPH method and in the order of 42.17 ± 7.46 and 79.77 ± 6.91 *μ*g/mL by the FRAP method, for both E.PG and I.PG, respectively. Furthermore, the inhibition percentages of calcium oxalate crystallization are in the range of 98.11 ± 0.17 and 98.22 ± 0.71% against the nucleation and in the order of 88.98 ± 0.98 and 88.78 ± 2.48% against the aggregation, for E.PG and I.PG, respectively. These results prove the richness of the plant in bioactive compounds, offering an antioxidant and anticrystallization capacity; therefore, it can be used in the treatment and/or the prevention of stone formation.

## 1. Introduction

Urolithiasis is a severe pathology characterized by the formation of stones in the kidneys or in the urinary tract and can lead to kidney failure. It affects 4 to 20% of the population and the recurrence rate was estimated at 50% during the first 5 years with the predominance of calcium oxalate [1–6]. The crystals of the latter are found mainly in three different forms, calcium oxalate monohydrate (COM) or Whewellite, calcium oxalate dihydrate (COD) or Weddellite, and calcium oxalate trihydrate (COT) [7–9]. The “COM” is the most thermodynamically stable form and has a high affinity for renal tubular cells; consequently, it is the initiator of stone formation in the kidney [10, 11].

The process of calcium oxalate crystallization begins with increased urinary supersaturation [12], followed by nucleation and aggregation [12–14]. The crystals develop and accumulate with other crystals in solution and are finally retained and accumulated in the kidney [14–16]. The damage to the kidney membrane induced by oxalate is mediated by free radicals [17]; consequently it promotes the crystalline retention at the renal papillary surface, as well as the crystal nucleation at lower levels of supersaturation [18]. In addition, the antioxidants contribute importantly on the prevention of stone formation by maintaining the normal physiological concentration of the free radicals and provide the protection against the oxidative damage of endothelial cells [17].

The plant *Punica granatum* L. “Pomegranate” belongs to the family of Punicaceae, considered to be a Middle East descendent. It extends throughout the Mediterranean, China, India, Europe, North America, and South America [19–21]. It is a small tree or a large shrub, with obovate deciduous leaves with bright red flowers. The fruits are berries delimited by a pericarp, containing many seeds surrounded by a translucent juice bag called arils attached to the inside of the fruit by the mesocarp [19, 22–24]. Regarding the bioactive compounds of the plant, pomegranate fruit remains a more diverse source of bioactive phenolic compounds, particularly phenolic acids, flavonoids, anthocyanins, and tannins [22, 25–28]. The saturated and unsaturated fatty acids are also present mainly in the seeds [29, 30]. This richness proves the curative and preventive potential of the plant against the chronic diseases, as well as the multiple biological activities, such as antimicrobial, antioxidant, antidiarrhea, antitumor, antimetastasis, antiproliferative, anti-inflammatory, and antinociceptive ones. Moreover, it has an effect against brain's oxidative damage and prevents giardiasis and obesity [30–40]. In this context, the purpose of this study is the evaluation of the anticrystallization effect of calcium oxalate monohydrate and the antioxidant effect of the two extracts made from *Punica granatum* L. fruit peels (I.PG and E.PG) in addition to the demonstration of the relationship between these two activities, then dosing and identifying their chemical compounds using the colorimetric methods and Ultra Performance Liquid chromatography-photodiode-array-electrospray ionization-mass spectrometry (UPLC-PDA-ESI-MS).

## 2. Materials and Methods

### 2.1. Extraction

#### 2.1.1. Hydroethanolic Extract

The fruits of *Punica granatum* L have been harvested from the Taounate region (located in the north of Morocco, 92 km from the city of Fez, 34°32′09″N, 4°38′24″W), in November 2017. Taxonomic identification was performed by Prof. A. Bari, Department of Biology, Faculty of Sciences Dhar El-Mahraz, Sidi Mohammed Ben Abdellah University, Fez, Morocco. The peels have been dried in the dark at a room temperature of 25°C and crushed to obtain a fine fraction. For the preparation of the hydroalcoholic extract (E.PG) we followed the method of Kachkoul et al. [41], which consists in introducing 20 g of powder in the cellulosic cartridge; the latter is inserted into the extractor of the Soxhlet assembly surmounted by a refrigerant and 170 mL of n-hexane in the mounting flask, following boiling for 4 h at 65°C. The lipid extract is then recovered by removing the solvent using a rotary evaporator under vacuum [42, 43]. Then, a second hydroalcoholic extraction was carried out on the defatted mark in the same way as the first extraction using a mixture of ethanol/distilled water (80 : 20 v : v) for 4 h; the elimination of ethanol and water is also done using the Rotavapor.

#### 2.1.2. Infusion

The infusion was prepared by the method described by Jiménez-Zamora et al. [44] with some modifications. Briefly, 2 g of the fruit peel powder placed in 100 mL of boiling distilled water is allowed to be infused for 30 min and then filtered using a filter paper with a diameter of 1.6 *μ*m. The infusion (I.PG) was concentrated by Rotavapor and stored in a temperature of −4°C until use [41, 45].

### 2.2. Determination of Total Polyphenol, Flavonoid, Flavonol, and Anthocyanin Contents

The content of total polyphenols has been quantified by the method of Folin-Ciocalteu according to the protocol described by María et al. [46]. Flavonoids and flavonols have been determined with the colorimetric methods using aluminum chloride, according to the protocols described by Iqbal et al. [47] and Awah et al. [48]. The total anthocyanins have been dosed by the pH variation method described by Hosseinian et al. [49] and Bhat and Riar [50].

### 2.3. UPLC-PDA-ESI-MS Analysis

The chemical compounds of E.PG and I.PG have been identified by UPLC in reverse phase using a Waters ACQUITY SDS UPLC system with a Column type: ACQUITY UPLC® BEH C18 1.7 *μ*m, as followed in the method of Kachkoul et al. [45]. In this respect, the separation is carried out in gradient mode using acetonitrile (CH_3_CN) and formic acid (100 : 0.1 v : v) according to the following steps: (1) initial mobile phase including 10% of CH_3_CN, (2) linear increase from 10% to 25% of CH_3_CN (0–18 min), (3) 25% of CH_3_CN (18–21 min), (4) from 25% to 50% of CH_3_CN (21–28 min), (5) from 50% to 100% of CH_3_CN (28- 29 min), and (6) 100% of CH_3_CN (29-30 min); then the percentage of CH_3_CN has been reduced to 10% (30–37 min), which allowed a stabilization time of 7 min before injection of the next sample. 4 *μ*L of the sample was injected and the flow rate has been set to 0.4 ml·min^−1^. UPLC was connected to a PDA diode array detector (Waters ACQUITY) with a detection wavelength range between 190 and 500 nm and an ion trap mass spectrometer (SYNAPT-G2#UCA014) equipped with an electrospray interface (ESI). Further, the scan range has been set to *m*/*z* which ranges from 50 to 1500 with a scan time of 0.3 s. Hence, the conditions of the ESI were as follows: there has been negative mode and temperature of the source and the desolvation gas was 120°C and 450°C. The flow rate of the cone, desolvation gas (Helium), and the collision (He) is 800 L/h, 20 L/h, and 0.01 mL/min, respectively.

### 2.4. Antioxidant Activity

#### 2.4.1. DPPH (2,2-diphenyl-1-picrylhydrazyl) Method

The antiradical power of the extracts E.PG and I.PG has been tested by the method of DPPH (1,1-diphenyl-2-picrylhydrazyl) as indicated in the protocol described by Wang et al. [51] with small modifications. In fact, a solution of DPPH has been prepared by the solubilization of 4 mg of this latter in 100 mL of methanol. 750 *μ*L of extracts with different concentrations ranging from 0.1 to 2 mg/mL and ascorbic acid (vitamin C (Vit.C)) with (0.01 to 2 mg/mL) are introduced into test tubes. Then 1.5 mL of the DPPH solution was added. After incubation for 30 min in the dark and at room temperature, the absorbance (*A*) has been measured at *λ* = 517 nm. The inhibition percentages have been calculated by the following relationship [45]:

(1) $$\begin{array}{c} \%\text{ antioxidant activity}=\;\frac{A\text{ control}–A\text{ simple}}{A\text{ control}}*100. \end{array}$$

#### 2.4.2. Ferric Reducing Antioxidant Power (FRAP) Method

The reducing power of the plant extracts has been determined according to the method of Oyaizu [52], which consists in mixing 2.5 mL of E.PG and I.PG extracts at different concentrations ranging from 0.1 to 2 mg/mL solubilized in distilled water with 2.5 mL of phosphate buffer (0.2 mol/L, pH 6.6) and 2.5 mL of potassium ferricyanide [K3Fe (CN) 6] (1%). To add, the mixture has been incubated at 50°C for 20 min. A volume of 2.5 mL of trichloroacetic acid (10%) has been added to the mixture and then centrifuged at 3000 rpm for 10 min. An aliquot of 2.5 mL of the upper layer of the solution was mixed with 2.5 mL of distilled water and 0.5 mL of FeCl_3_ (0.1%). Absorbance has been measured at *λ* = 700 nm. Ascorbic acid was used as a control [45, 51, 53].

### 2.5. Inhibition of the Calcium Oxalate's Crystallization

The anticrystallization effect of the plant extracts has been studied according to the protocol described by Hess et al. [54] and repeated by Kachkoul et al. [41, 55]. The stock solutions of CaCl_2_ : 2H_2_O [15 mM] and Na_2_C_2_O_4_ [1.5 mM] containing NaCl [200 mM] have been prepared, to which 10 mM sodium acetate was added to adjust the pH value to 5.7. Thereafter, the solutions have been filtered through 0.22 *μ*m pore diameter filters and then warmed to 37°C before being used. For crystallization experiments without inhibitor, 1 mL of the CaCl_2_ : 2H_2_O solution and 1 mL of the distilled water have been transferred to a quartz cuvette optical path 10 mm; then an identical volume of the Na_2_C_2_O_4_ solution has been added to trigger the crystals' formation. Then, the temporal measurements of the optical density “OD” have been recorded every 15 seconds for a period of one hour, using a UV-Visible spectrophotometer at the wavelength *λ* equal to 620 nm, which corresponds to the crystal detection. Similarly, the tests with inhibitors have been performed with several concentrations of 0.5, 1, and 2 g/L of I.PG and E.PG extracts and in the same way as the tests without inhibitors, by replacing the distilled water with the extracts. Besides, the slopes of the nucleation phase “SN” and aggregation “SA” have been calculated using linear regression analysis, and the percentages of inhibitions have been calculated by the relation of Hess et al. [54, 56]

(2) $$\begin{array}{c} \%\text{ nucleation inhibition}=1-\frac{\text{SN}i}{\text{SN}s}*100,\\ \%\text{ aggregation inhibition}=1-\frac{\text{SA}i}{\text{SA}s}*100, \end{array}$$

where “*i*” and “*s*” represent tests with and without inhibitors, respectively. The correlation coefficient (*R*) and the coefficient of variation (CV) are calculated to verify the validity of our results.

### 2.6. Characterization of Crystals

The characterization of the crystals has been performed using an optical microscope, and the determination of the chemical composition has been made by Fourier Transform Infrared Spectroscopy (FT-IR) between 4000 and 400 cm^−1^ with a resolution of 4 cm^−1^, on crystals synthesized in the absence and in the presence of inhibitors.

### 2.7. Statistical Analysis

The results were expressed as mean ± SD of the three repetitions; the statistical analyses have been carried out by ANOVA One-Way method followed by Tukey's multiple comparison test and correlation of Pearson's using graphPad Prism7; *p* < 0.05 is considered as significant.

## 3. Results and Discussion

### 3.1. Content of Total Polyphenols, Flavonoids, Flavonols, and Anthocyanins

**Figure 1:**
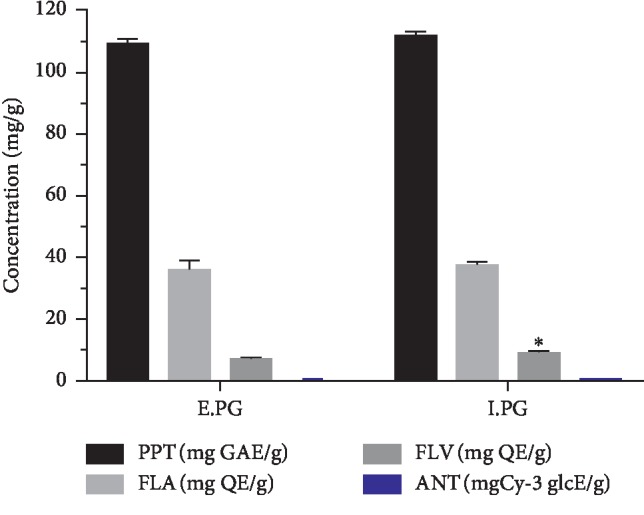
Content of total polyphenols, flavonoids, flavonols, and anthocyanins in the extracts of E.PG and I.PG. Values are expressed as mean ± SD (*n* = 3). ^*∗*^*p* < 0.05 vs. E.PG. E.PG: hydroalcoholic extract of *Punica granatum* L.; I.PG: infusion of *Punica granatum* L.; PPT: total polyphenols; FLA: flavonoids; FLV: flavonols; ANT: anthocyanins.

The contents of Total Polyphenol, Flavonoids, Flavonols, and Anthocyanins of the two extracts are illustrated in Figure 1.

As shown in Figure 1, there is no significant variation between the two extracts of the plant for the different chemical families assayed excepted flavonols (*p* < 0.05); the results of the quantification reveal the richness of the pomegranate fruit peel by the total polyphenols, with contents of 109.44 ± 1.37 and 111.92 ± 1.42 mg GAE/g for E.PG and I.PG, respectively. With regard to flavonoids and flavonols, which are by definition subclasses of polyphenols, the values obtained are in the order of 36.05 ± 2.95 and 37.64 ± 0.91 mg EQ/g for the first class and in the order of 7.17 ± 0.49 and 9.08 ± 0.33 mg EQ/g for the second class, respectively, for E.PG and I.PG. However, anthocyanins are present in low levels with values of the order of 0.47 ± 0.096 and 0.67 ± 0.16 mgCy-3 glcE/g for E.PG and I.PG, respectively. The comparison of these outcomes found in this study with the literature shows that the contents of these extracts far exceed those found by Romeo et al. [57], who reported concentrations of 66.97 ± 0.67 g GAE/kg for polyphenol and 21.64 ± 0.03 mg mgCy-3 glcE/kg for anthocyanins. On the other hand, Derakhshan et al. [58] found higher values in three Iranian varieties (Natanz, Shahreza, and Doorak) for total polyphenols and flavonols, but the flavonoid values remain comparable with this work.

### 3.2. UPLC-PDA-ESI-MS Analysis

**Figure 2:**
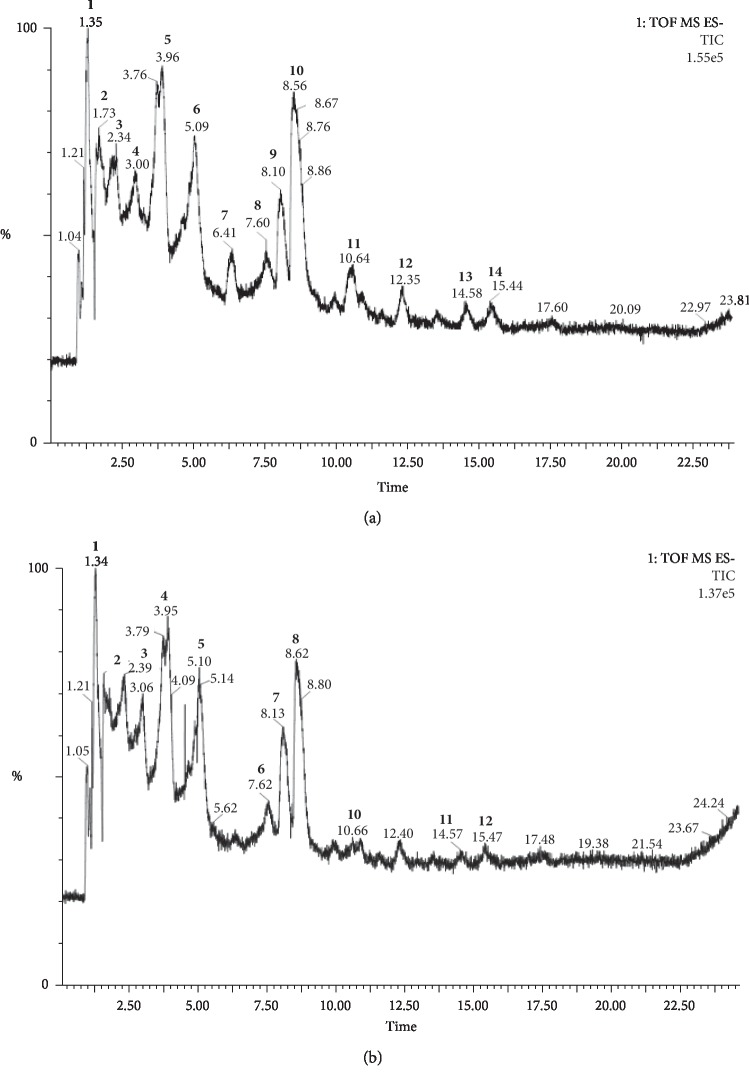
UPLC chromatograms of plant extracts; (a) E.PG and (b) I.PG. E.PG: hydroalcoholic extract of *Punica granatum* L.; I.PG: infusion of *Punica granatum* L. The bold numbers marked the peaks, whose names are shown in Tables 1 and 2.

The determination of the likely chemical composition in the E.PG and I.PG extracts of the pomegranate fruit peel was based on comparing the absorbance and the MS spectrum obtained by UPLC-PDA-ESI-MS with the literature data. The identification results and the chromatographic profile for these two extracts are shown in Figure 2 and in Tables 1 and 2.

**Figure 3:**
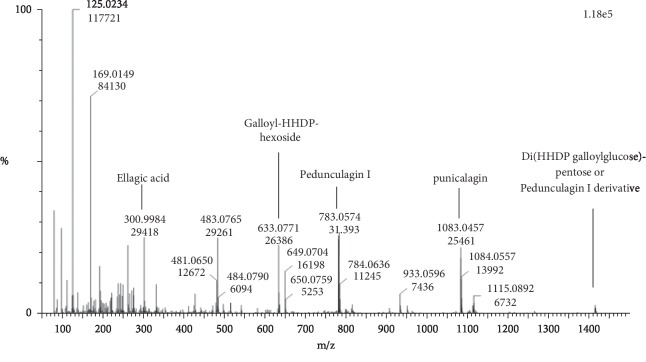
MS spectrum of the molecule Di(HHDP-galloyl-glucose)-pentose or pedunculagin I derivative present in both plant extracts.

The chemical composition of the E.PG extract (Table 1) is dominated by phenolic acid derivatives mainly the Ellagitannins. UPLC chromatogram (Figure 2(a)) of this extract illustrates the presence of 14 peaks. The interpretation of their MS spectra reveals the presence of the following compounds: peaks **1** and **2** are characterized by the presence of the pseudomolecular ion [MH]^−^ at *m*/*z* 1083; main fragments have been observed at *m*/*z* 781 and 301, indicating the loss of ellagic acid and corresponding to the punicalagin compound [65]. Peaks **3** and **4** with an [M-H]^−^ ion at *m*/*z* 1415 represent the molecule of Di(HHDP-galloyl-glucose)-pentose [27] or pedunculagin I derivative [60]; this compound produces the fragments at *m*/*z* 1083 which corresponds to punicalagin, at *m*/*z* 633 and 301, which are characteristics of galloyl-HHDP-hexoside and ellagic acid, respectively (Figure 3) [25, 64, 66].

Peak **5** is tentative identified as Granatin A (ellagic acid derivative) or lagerstannin A (bis-HHDP-gluconic acid) [62], with the pseudomolecular ion [M-H]^−^ at *m*/*z* 799 and the presence of fragment at *m*/*z* 301 which illustrates the ellagic acid molecule. Peak **7** revealed a molecular ion [M-H]^−^ at *m*/*z* 785 typical of the pedunculagin II molecule (Digalloyl-HHDP-hexoside). Peaks **8** and **9** are identified as Granatin B (galloyl-HHDP-DHHDP-hexoside) or pedunculagin I derivative; these two peaks are characterized by the presence of a pseudomolecular ion [M-H]^−^ at *m*/*z* 951 [22, 60–62], and the fragments have been detected at *m*/*z* 933 that represents the loss of a molecule of water ([M-H- H_2_O]^−^) [63], at *m*/*z* 783, which can illustrate the loss of the gallic acid molecule ([M-H- acide gallique]^−^) or the pedunculagin I molecule, and at *m*/*z* 301, which typically expresses the fragment of ellagic acid. The peak **11** displays a pseudomolecular ion [M-H]^−^ at *m*/*z* 965 and a fragment at *m*/*z* 301 can illustrate the Castalagin derivative [22, 62, 64]. The peak **14** was identified as Vanillic acid-hexoside derivative and exhibits a molecular ion [MH]^−^ at *m*/*z* 659 and a fragment at *m*/*z* 329; this latter illustrates the molecule of vanillic-hexoside acid. Regarding the infusion, the UPLC chromatogram (Figure 2(b)) shows the presence of 12 peaks; their characterization (Table 2) reveals a similarity with the hydroalcoholic extract, with the absence of the vanillic acid-hexoside derivative molecule and the presence of the molecule punigluconin; this latter has not been detected in the first extract.

### 3.3. Antioxidant Activity

**Figure 4:**
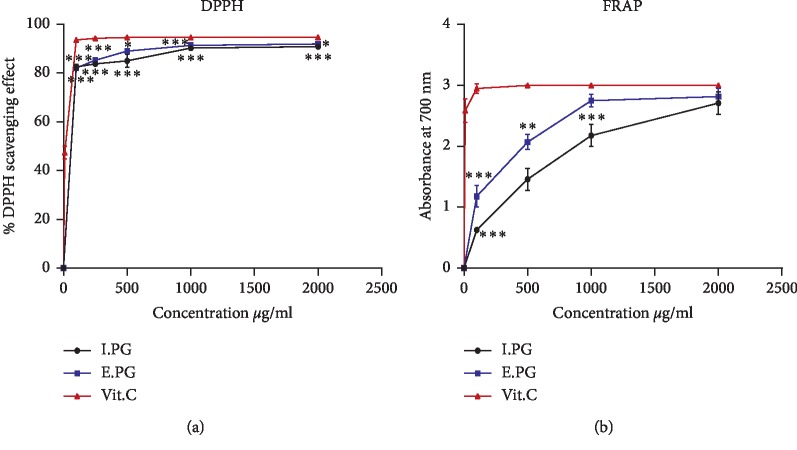
Antioxidant effect of I.PG I.PG and Vit.C. (a) Percent inhibition of free DPPH. (b) Ferric reducing power. Values are expressed as mean ± SD (*n* = 3). E.PG: hydroalcoholic extract of *Punica granatum* L.; I.PG: infusion of *Punica granatum* L.; Vit.C : vitamin C. ^*∗*^*p* < 0.05; ^*∗∗*^*p* < 0.01; ^*∗∗∗*^*p* < 0.005 vs. Vit.C group.

The antioxidant activity has been verified in vitro by DPPH and FRAP methods. In order to judge the importance of the antiradical effect of the E.PG and I.PG extracts, a positive control has been studied using vitamin C (Vit.C) known for its antioxidant power. In fact, percent inhibition and the median inhibitory concentration (IC_50_) are represented in Figure 4 and Table 3.

According to Figure 4(a), corresponding to the reducing activity of the DPPH radical, the inhibition rate increases rapidly in function of the concentration increase for the three products, ended by stabilization at concentrations of 100 *μ*g/mL for Vit.C and 1000 *μ*g/mL for both extracts. The maximum inhibition percentages that correspond to the concentration of 2 mg/ml are almost similar for both extracts I.PG and E.PG, with a superiority for the second; the values found are in the order of 90.86 ± 0.55 (*p* < 0.05 vs. Vit.C) and 91.93 ± 0.43% (*p* < 0.05 vs. Vit.C) for I.PG and E.PG, respectively. These percentages are close to the positive control (Vit.C) which has a value of 94.74 ± 0.20%. In addition, the median inhibitory concentration (IC_50_) also exhibits a similarity between the two extracts with values of 60.87 ± 0.27 *μ*g/mL (*p* < 0.05 vs. Vit.C) and 59.91 ± 0.83 *μ*g/mL (*p* < 0.05 vs. Vit.C) for I.PG and E.PG, respectively, against 10.57 ± 0.61 *μ*g/mL for Vit.C. These data prove the effectiveness of the plant extracts in the process of free radical removal by transforming them into more stable products at low concentrations. In fact, the mechanism of action involves donating hydrogen to a free radical, thereby reducing it to a nonreactive species. The addition of hydrogen removes the characteristic of odd electrons, which are responsible for radical reactivity [51].

The ferric reducing antioxidant power (FRAP) of the two extracts exhibits a great deal of capacity in reducing the Fe3^+^/ferricyanide complex to a ferrous Fe2^+^ form by the donation of an electron. As shown in Figure 4(b), the absorbance increases correlatively as a function of the concentration and reaches maximum values in the order of 2.70 ± 0.19 and 2.81 ± 0.14 at concentration of 2 mg/ml for I.PG and E.PG, respectively, whereas, for Vit.C, the absorbance increases rapidly and takes a value of 2.95 ± 0.078 at a lower concentration (0.1 mg/mL). However, the IC_50_ values which represent the concentration of the studied solutions at 0.5 of absorption prove the efficacy of the E.PG compared to the I.PG, with values of42.17 ± 7.46 *μ*g/mL (*p* < 0.05 vs. Vit.C) and 79.77 ± 6.91 *μ*g/mL (*p* < 0.05 vs. Vit.C), respectively, while the solution of Vit.C reveals a value of 1.94 ± 0.14 *μ*g/mL. In this respect, the DPPH method outcomes in this work are approximating those reported by Abid et al. [64] who worked on different extracts of the Tunisian varieties (Acid, Gabsi, Nebli, and Tounsi) and provide reduction values between 71.11% and 97.82%, as well as the works of Šavikin et al. [67] and Pagliarulo et al. [68]. Moreover, this antiradical efficacy of these extracts is probably due to the presence of ellagic acid derivatives such as punicalagin. The ability of this molecule in free radical scavenging has been demonstrated by Aguilar-Zárate et al. [69] and the values found are more than 90% at a concentration of 250 *μ*g/ml and an IC_50_ of 109.53 *μ*g/mL.

### 3.4. Inhibition of the Calcium Oxalate's Crystallization

The preventive effect of the E.PG and I.PG extracts of the plant *Punica granatum* L. against the calcium oxalate's crystallization has been studied in vitro by the turbidimetric model in absence (SI) and presence of inhibitors. This model allows following the steps of crystallization, in particular, the nucleation and aggregation stages. In fact, the inhibition percentages of the last two have been calculated from the comparison of the slopes in the presence of the extracts with that without inhibitor and the outcomes are displayed in Table 4.

**Figure 5:**
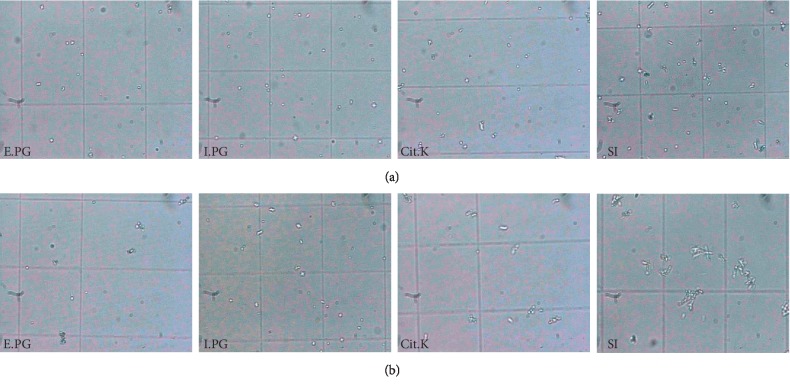
Crystals of calcium oxalate in the stages of nucleation (a) and aggregation (b). E.PG: hydroalcoholic extract of *Punica granatum* L.; I.PG: infusion of *Punica granatum* L.; Cit.K: potassium citrate.

The analysis of the data in Table 4 shows a high efficiency of the two plant extracts in a dose-dependent manner in inhibiting the nucleation of calcium oxalate crystals. In fact, the inhibition percentages are in order of 93.38 ± 0.09, 94.97 ± 0.21, and 98.11 ± 0.17% (*P* < 0.05 vs. Cit.K) (*R* > 0.95, CV < 10%) for E.PG at concentrations of 0.5, 1, and 2 g/L, respectively, and in the order of 93.01 ± 1.22% (*P* < 0.05 vs. Cit.K), 97.59 ± 0.30, and 98.22 ± 0.71% (*R* > 0.95, CV < 10%) for I.PG at concentrations of 0.5, 1, and 2 g/L, respectively, whereas, for potassium citrate (Cit.K) taken as a positive control, the values found are in the order of 96.52 ± 0.01, 97.01 ± 0.06, and 97.37 ± 0.16% (*R* > 0.95, CV < 10%) at concentrations of 0.5, 1, and 2 g/L, respectively [55]. Nevertheless, the analysis of the images taken during this phase (Figure 5(a)) displays that the number and the size become less important in presence of the extracts of E.PG, I.PG, and Cit.K solution. This reflects the results of the turbidity model and confirms the effect of the plant extracts in nucleation inhibition, which appears to be more effective than the Cit.K solution.

Regarding the aggregation phase (Table 4), a similarity of inhibition rate has been observed in both extracts of E.PG and I.PG. Indeed, the inhibition rate of the latter is in the order of 72.28 ± 1.30, 84.66 ± 0.82, and 88.78 ± 2.48% (*p* < 0.05 vs. Cit.K) (*R* > 0.95, CV < 10%) for concentrations of 0.5, 1, and 2 g/L, respectively; also, the extract E.PG reveals values of 81.50 ± 0.99, 83.08 ± 1.54, and 88.98 ± 0.98% (*p* < 0.05 vs. Cit.K) (*R* > 0.95, CV < 10%), respectively, for concentrations of 0.5, 1, and 2 g/L, while the Cit.K solution shows a less important effect than the two extracts, with inhibition rate of 68.25 ± 2.4, 69.70 ± 2.85, and 77.12 ± 2.16% (*R* > 0.95, CV < 10%) for the same concentrations [55]. In addition, the image analysis relating to this phase (Figure 5(b)) exhibits the presence of larger and more numerous aggregates in the absence of inhibitor, while the size and the number of crystals become less important in the case of the treatment, especiallyE.PG and the I.PG. Moreover, the results of this study are more important than our subsequent results on crude methanolic extracts of the same plant and those of *Ammi visnaga* [55], as well as the hydroethanolic extract and the infusion of *A. unedo* leaves [41]. Similarly, they far exceed those of Sharma et al. [70] who worked on the aqueous extract of the *Chenopodium album* leaves and Cystone.

These outcomes can be explained by the presence of polar compounds in the plant extracts such as polyphenols, flavonoid, and tannins (derived from gallic acids) (Figure 1 and Tables 1 and 2). These compounds probably act through their ability of forming soluble chemical species that will reduce the risk of crystallization or by adsorption on the surface of crystals due to their numerous anionic charges and consequently an inhibition of crystal growth and aggregation. In addition, their fixations on the crystallites lead to an alteration of electrical attraction's phenomena between the atoms situated on the surface of the crystal and the ions present in the solution [12]. This mechanism was partially demonstrated by Noorafshan et al. [71] who reported that Diosmin which is a flavonoid prevents CaC_2_O_4_ deposition on crystals.

### 3.5. Crystal's Characterization by Fourier Transform Infrared Spectroscopy (FT-IR)

**Figure 6:**
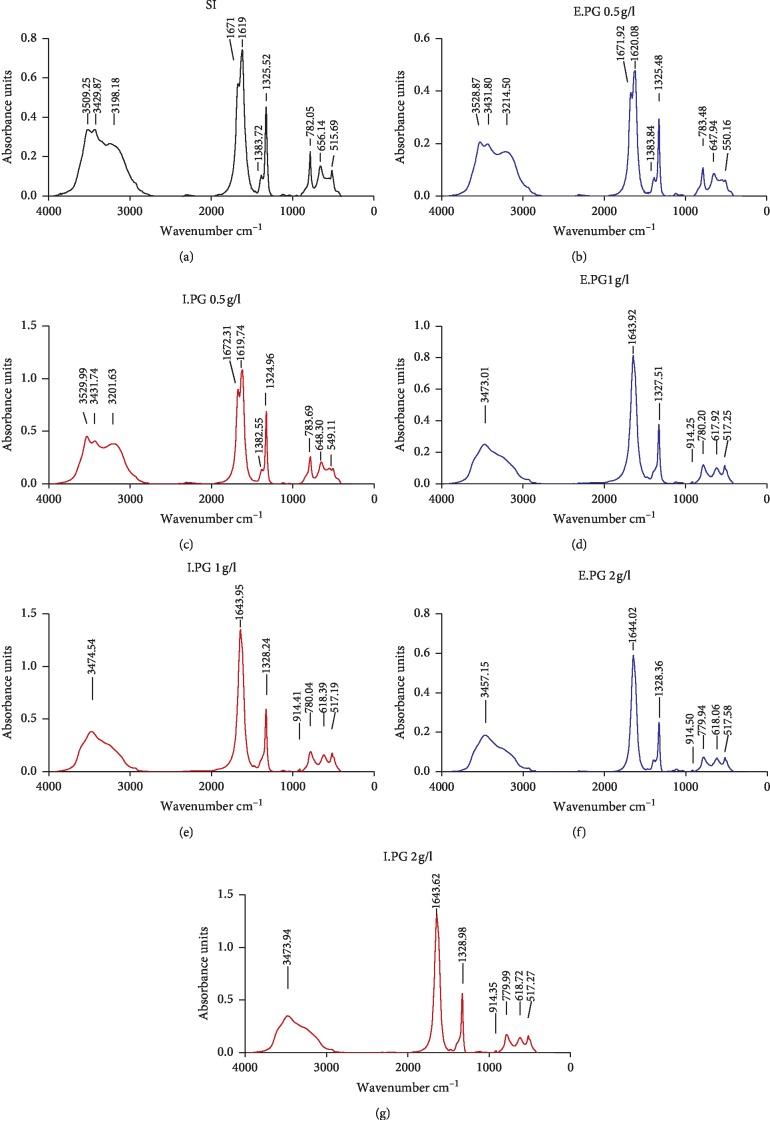
FT-IR spectra of the calcium oxalate crystals in absence (IS) and presence of the different concentrations of E.PG and I.PG. SI: absence of inhibitor; E.PG: hydroalcoholic extract of *Punica granatum* L.; I.PG: infusion of *Punica granatum* L.

The determination of the crystals synthesized chemical constitution in the absence and presence of the different concentrations plant extracts, as well as their transformations in terms of their types, has been carried out by the Fourier transform infrared spectroscopy (FT-IR). The spectra obtained are illustrated in Figure 6.

The analysis of the spectra displayed in Figure 6 exhibits that, in the absence of inhibitor (SI), the presence of two bands at 3509 and 3429 cm^−1^ critical to the OH stretching of the water, an out-of-plane C-O deformation band, and another O-C-O plane bending have been observed at 782 and 515 cm^−1^, respectively [72, 73]. While absorption bands have been observed at 1671/1619 cm^−1^ and 1383/1325 cm^−1^, the first are attributed to the stretching band antisymmetric carbonyls (vas (COO^−^)) and the second are compatible with the symmetrical stretching band of the metal-carboxylate (vs (COO^−^)); these bands are corresponding to crystals of COT [73–76]. Moreover, in the presence of E.PG and I.PG extracts, at a concentration of 0.5 g/L, multiple bands have been observed between 3200 and 3530 cm^−1^ critical to the OH stretching of the water, as well as the presence of a pointed bending band in the plane at 783 cm^−1^ corresponding to the bands of COM crystals [74]. The existence of bands at 648 ± 1 and 550 ± 1 cm^−1^ and absorption bands (vas (COO^−^)) at 1672/1620 cm^−1^ and (vs (COO^−^)) at 1383/1325 cm^−1^ asserts the formation of COT crystals [74, 75], so that it meant a mixture of COM + COT crystals. Besides that, in the presence of E.PG and I.PG at concentrations of 1 and 2 g/L, a single peak of absorption between 3474 and 3457 cm^−1^ has been founded, affirming the presence the COD crystals. In [77], a bending band out of the water at 780 cm^−1^ [78] and also two absorption bands have been observed at 1644 cm^−1^ for (vas (COO^−^)) and 1328 cm^−1^ for (vs (COO^−^)) attesting the formation of COD [74, 79].

### 3.6. Correlation between Phenolic Compounds and Antioxidant and Anti-crystallization Activities

The relationship between phenolic compounds and antioxidant and anticrystallization activities has been investigated by Pearson's correlation method, and the results are displayed in Tables 5 and 6.

The analysis of Pearson's correlation of I.PG and E.PG extracts (Tables 5 and 6) reveals a similarity of the correlation behavior in both plant extracts. Indeed, these data show a significant relationship between the DPPH radical reducing activity and the anticrystallization activity of calcium oxalate in the nucleation stage [(*R* = 1, *p* < 0.005 for I.PG) (*R* = 0.998, *p* < 0.01 for E.PG)] and in the aggregation stage [(*R* = .0.993; *p* < 0.01)) (*R* = 1, *p* < 0.005 for E.PG)]. A significant correlation between these last two stages and the ferric reducing power (FRAP) has been observed with [(*R* = 0.951, *p* < 0.05 for I.PG)) (*R* = 0.977, *p* < 0.05 for E.PG) for the nucleation and [(*R* = 0.977, *p* < 0.05 for I.PG)) (*R* = 0.972, *p* < 0.05 for E.PG)] for aggregation. Also, there wasa strong correlation between the two stages of calcium oxalate crystallization [(*R* = 0.994, *p* < 0.001 for I.PG) (*R* = 0.999, *p* < 0.005) for E.PG]. These outcomes can be explained in the way that the process of preventing the oxalate complexation with calcium and the inhibition of CaC_2_O_4_ deposit formation on crystals on the one hand and the process of the free radicals' removal by active ingredients of the extracts on the other hand act in a dependent manner and probably share the same reaction mechanism. Moreover, a strong correlation between the ferric reducing power and the phenolic compounds has been observed [(0.917 ≤ *R* ≤ 0.977, *p* < 0.05 for I.PG)) (0.845 ≤ *R* ≤ 0.977, *p* < 0.05 for E.PG)]; it suggests that the ability of the extracts in reducing the ferricyanide (Fe3^+^) complex to form more stable ferrous (Fe2^+^) was directly proportional to the phenolic compounds concentration [80].

## 4. Conclusion

This work highlights the high efficiency of the two fruit peel extracts of *Punica granatum* L. plant in the inhibition of the DPPH radical and a great reducing power of the Fe3^+^/ferricyanide complex; this capacity has been demonstrated by low IC_50_ values which are 59.91 ± 0.83 and 42.17 ± 7.46 *μ*g/mL for the DPPH and FRAP methods, respectively. Moreover, a large calcium oxalate anticrystallization effect has been revealed with inhibition percentages of 98.22 ± 0.71 and 88.98 ± 0.98% for the nucleation and the aggregation stages, respectively. The analysis by Fourier Transform Infrared Spectroscopy (FT-IR) displays the formation of COD crystals in the presence of extracts at concentrations greater than 1 g/L; these crystals have a low affinity for renal tubular cells. Yet, in the absence and presence of the extracts at low concentrations, there is formation of COM crystals and mixture of COT + COM. In parallel, a significant correlation between the anticrystallization and antioxidant activities has been revealed. However, these excellent activities of the plant can be attributed to higher levels of phenolic compounds quantified and identified by UPLC-PDA-MS, suggesting that it may have prospective use as preventive and/or therapeutic agents for urolithiasis and oxidative stress-related diseases.

## Figures and Tables

**Table 1:** Chemical compounds identified in hydroalcoholic extract of *Punica granatum* L.

Peak	RT (min)	[M-H]^−^(*m*/*z*)	PDA	Fragment ions (*m*/*z*)	Putative identity	Reference
**1**	1.35	1083	226/257/378	1083/781/481/301	Punicalagin	[26, 57, 59]
**2**	1.73	1083	216/261/378	1083/781/633/483/301	Punicalagin	[26, 57, 59]
**3**	2.34	1415	220/257/377	1415/1083/783/633/425/301	Di(HHDP-galloyl-glucose)-pentose Pedunculagin I derivative	[22, 27, 60]
**4**	3.00	1415	219/257/377	1415/1083/783/633/483/301	Di(HHDP-galloyl-glucose)-pentose Pedunculagin I derivative	[22, 27, 60]
**5**	3.96	799		799/301/247	Granatin A ellagic acid derivative	[22, 61, 62]
**6**	5.09	927	252/361	927/813/633/463/300	Unknown	
**7**	6.41	785		785/601/247	Pedunculagin II (digalloyl-HHDP-hexoside)	[22, 62, 63]
**8**	7.6	951	220/255	951/933/783/433/301/216	Granatin B (galloyl-HHDP-DHHDP-hexoside)/pedunculagin I derivative	[22, 60–62]
**9**	8.10	951	197/252/361	951/867/783/433/301/216	Granatin B (galloyl-HHDP-DHHDP-hexoside)/pedunculagin I derivative	[22, 57, 61, 62]
**10**	8.56	301	252/367	301	Ellagic acid	[22, 26, 62]
**11**	10.64	965		965/951/319/301/273/217	Castalagin der	[22, 62, 64]
**12**	12.35	917		917/703/585/425/216	Unknown	
**13**	14.58	907		907/633/447/292/216	Unknown	
**14**	15.44	659		659/390/329/216	Vanillic acid-hex der	[22]

**Table 2:** Chemical compounds identified in infusion of *Punica granatum* L.

Peak	RT (min)	[M-H]^−^(*m*/*z*)	PDA	Fragment ions (*m*/*z*)	Putative identity	Reference
**1**	1.34	1083	228/257	1083/781/633/481/301	Punicalagin	[26, 57, 59]
**2**	2.39	1265	377/257	1265/1083/783/633/541/425/301/275	Pedunculagin I derivative	[22]
**3**	3.06	1415	220/257/378	1415/1083/753/633/483/301/275	Di(HHDP-galloyl-glucose)-pentose	[27]
**4**	3.95	801		801/479/301/247	Digalloyl-HHDP-glucose (punigluconin)	[22, 62, 63]
**5**	5.10	927	252/361	927/813/633/463/301	Unknown	
**6**	7.62	951		951/783/433/301/217	Granatin B (galloyl-HHDP-DHHDP-hexoside)	[22, 61, 62]
**7**	8.13	951	196/252/361	951/447/433/301/217	Granatin B (galloyl-HHDP-DHHDP-hexoside)	[22, 61, 62]
**8**	8.62	301	252/367	301	Ellagic acid	[22, 26, 62]
**9**	10.94	965		965/477/301/292/217	Castalagin der	[22, 62]
**10**	12.40	585		585/451/292/217	Unknown	
**11**	14.57	907		907/391/329/217	Unknown	
**12**	15.47	659		659/391/329/217	Unknown	

**Table 3:** IC_50_ values of E.PG, I.PG, and Vit.C obtained with the DPPH and FRAP method.

Extract	IC_50_ value (*μ*g/ml)	
	DPPH	FRAP
E.PG	60.87 ± 0.27^*∗∗∗*^	42.17 ± 7.46^*∗*^
I.PG	59.91 ± 0.83^*∗∗∗*^	79.77 ± 6.91^*∗∗*^
V.C	10.57 ± 0.61	1.94 ± 0.14

Values are expressed as mean ± SD (*n* = 3). E.PG: hydroalcoholic extract of *Punica granatum* L.; I.PG: infusion of *Punica granatum* L.; Vit.C: vitamin C. ^*∗*^*p* < 0.05; ^*∗∗*^*p* < 0.01; ^*∗∗∗*^*p* < 0.005 vs. Vit.C group.

**Table 4:** Percent inhibition of nucleation and aggregation in the presence of plant extracts and Cit.K solution.

Concentration g/l	Nucleation %			Aggregation %		
	E.PG	I.PG	CIT.K §	E.PG	I.PG	Cit.K §
**0.5**	93.38 ± 0.09^*∗*a*#*^	93.01 ± 1.22^*∗*a*#*^	96.52 ± 0.01^a#^	81.50 ± 0.99^*∗∗*a*#*^	72.28 ± 1.30^*∗*a*#*^	68.25 ± 2.4^a#^
**1**	94.97 ± 0.21^*∗∗*a*#*^	97.59 ± 0.30^a#^	97.01 ± 0.06^a#^	83.08 ± 1.54^*∗∗*a*#*^	84.66 ± 0.82^*∗∗*a*#*^	69.70 ± 2.85^a#^
**2**	98.11 ± 0.17^*∗*a*#*^	98.22 ± 0.71^a#^	97.37 ± 0.16^a#^	88.98 ± 0.98^*∗*a*#*^	88.78 ± 2.48^*∗*a*#*^	77.12 ± 2.16^a#^

Values are expressed as mean ± SD (*n* = 3). E.PG: hydroalcoholic extract of *Punica granatum* L.; I.PG: infusion of *Punica granatum* L.; Cit.K: potassium citrate. §: Kachkoul et al. [55]. ^*∗*^*p* < 0.05^*∗∗*^*p* < 0.01 vs. Vit.C. ^a^*R* > 0.95. ^#^CV < 10%.

**Table 5:** Correlation between phenolic compounds and antioxidant and anticrystallization activity of I.PG extract.

	DPPH	FRAP	PPT	FLA	FLV	ANT	% N	% A
DPPH	1							
FRAP	0.694	1						
PPT	0.484	0.917^*∗∗*^	1					
FLA	0.484	0.917^*∗∗*^	1^*∗∗∗*^	1				
FLV	0.484	0.917^*∗∗*^	1^*∗∗∗*^	1^*∗∗∗*^	1			
ANT	0.484	0.917^*∗∗*^	1^*∗∗∗*^	1^*∗∗∗*^	1^*∗∗∗*^	1		
% N	1^*∗∗∗*^	0.951^*∗*^	0.711	0.711	0.711	0.711	1	
% A	0.993^*∗∗*^	0.977^*∗*^	0.787	0.787	0.787	0.787	0.994^*∗∗*^	1

I.PG: infusion of *Punica granatum* L.; PPT: total polyphenols; FLA: flavonoids; FLV: flavonols; ANT: anthocyanins; % N:% nucleation inhibition; %A: % aggregation inhibition. ^*∗*^Correlation is significant at *p* < 0.05. ^*∗∗*^Correlation is significant at *p* < 0.01. ^*∗∗∗*^Correlation is significant at *p* < 0.005. ^*∗∗∗∗*^Correlation is significant at *p* < 0.001.

**Table 6:** Correlation between phenolic compounds and antioxidant and anticrystallization activity of E.PG extract.

	DPPH	FRAP	PPT	FLA	FLV	ANT	% N	% A
DPPH	1							
FRAP	0.790	1						
PPT	0.490	0.845^*∗*^	1					
FLA	0.490	0.845^*∗*^	1^*∗∗∗*^	1				
FLV	0.490	0.845^*∗*^	1^*∗∗∗*^	0.999^*∗∗∗*^	1			
ANT	0.490	0.845^*∗*^	1^*∗∗∗∗*^	1^*∗∗∗*^	1^*∗∗∗*^	1		
% N	0.998^*∗∗*^	0.977^*∗*^	0.736	0.736	0.736	0.736	1	
% A	1^*∗∗∗*^	0.972^*∗*^	0.713	0.713	0.713	0.713	0.999^*∗∗∗*^	1

E.PG: hydroalcoholic extract of *Punica granatum* L.; PPT: total polyphenols; FLA: flavonoids; FLV: flavonols; ANT: anthocyanins; % N:% nucleation inhibition; %A: % aggregation inhibition. ^*∗*^Correlation is significant at *p* < 0.05. ^*∗∗*^Correlation is significant at *p* < 0.01. ^*∗∗∗*^Correlation is significant at *p* < 0.005. ^*∗∗∗∗*^Correlation is significant at *p* < 0.001.

## Data Availability

The data used to support the findings of this study are available from the corresponding author upon request.
